# Salvianolic Acid B Promotes the Survival of Random-Pattern Skin Flaps in Rats by Inducing Autophagy

**DOI:** 10.3389/fphar.2018.01178

**Published:** 2018-10-23

**Authors:** Jinti Lin, Renjin Lin, Shihen Li, Hongqiang Wu, Jian Ding, Guangheng Xiang, Shi Li, Yiru Wang, Dingsheng Lin, Weiyang Gao, Jianzhong Kong, Huazi Xu, Kailiang Zhou

**Affiliations:** ^1^Department of Orthopaedics, The Second Affiliated Hospital and Yuying Children’s Hospital of Wenzhou Medical University, Wenzhou, China; ^2^Zhejiang Provincial Key Laboratory of Orthopaedics, Wenzhou, China; ^3^The Second Clinical Medical College of Wenzhou Medical University, Wenzhou, China; ^4^Department of Neurology, Wenzhou Traditional Chinese Medicine Hospital, Wenzhou, China

**Keywords:** salvianolic acid B, random skin flap, autophagy, angiogenesis, apoptosis, oxidative stress

## Abstract

Random-pattern skin flap transplantation is frequently applied in plastic and reconstructive surgery. However, the distal part of the flap often suffers necrosis due to ischemia. In this study, the effects of salvianolic acid B (Sal B) on flap survival were evaluated, and the underlying mechanisms were investigated. Sal B improved the survival area, reduced tissue edema, and increased the number of microvessels in skin flaps after 7 days, whereas an autophagy inhibitor (3-methyladenine) reversed the Sal B-induced increase in flap viability. In addition, Sal B stimulated angiogenesis, inhibited apoptosis, reduced oxidative stress, and upregulated autophagy in areas of ischemia. Moreover, the effects of Sal B on angiogenesis, apoptosis, and oxidative stress were reversed by autophagy inhibition. Overall, our findings suggest that Sal B has pro-angiogenesis, anti-apoptosis, and anti-oxidative stress effects by stimulating autophagy, which enhances the survival of random-pattern skin flaps.

## Introduction

Random-pattern skin flaps are commonly used in plastic surgery to cover skin defects, which are caused by trauma, oncologic resection, or congenital conditions (Tanaka et al., 2003; Harder et al., 2008; Wang et al., 2014; Kanayama et al., 2017). However, necrosis of partial flaps, one of the most frequent post-operative complications, limits their clinical application, as it restricts the length-to-width ratio of flaps to 1.5 or 2:1 (Kailiang et al., 2016). The viability of random flaps depends on an adequate blood supply; thus, tissue necrosis in distal random flaps is typically due to a lack of angiogenesis (Stell, 1976). After establishing a random flap, a new vasoganglion starts to form from the pedicle bed of the flap toward the distal end. Therefore, angiogenesis is infrequent in the distal part of the flap (Saito et al., 2018). After vascular regeneration in a cutaneous flap, reperfusion and restoration of blood supply result in IRI by promoting necrosis (Lin et al., 2017). Oxidative stress and apoptosis are two major mechanisms of IRI and are key factors in the necrosis of random-pattern flaps (Deheng et al., 2016). Thus, ischemic necrosis of random skin flaps can be ameliorated by promoting angiogenesis and inhibiting oxidative stress and apoptosis.

Autophagy, the degradation of cytosolic macromolecules and organelles in lysosomes, is crucial in angiogenesis and IRI (Raben and Puertollano, 2016; Chen et al., 2017). Activation of autophagy up-regulates AKT phosphorylation in endothelial cells, leading to promotion of angiogenesis (Perez-Alvarez et al., 2018). Moreover, it alleviates cerebral IRI after inhibition of mammalian target of rapamycin complex-1 (mTOR1) (Perez-Alvarez et al., 2018). Interestingly, calcitriol enhances the viability of random flaps by activating autophagy, which alleviates IRI and promotes angiogenesis (Zhou et al., 2016; Chen et al., 2017). Therefore, an agent that promotes autophagy and angiogenesis and inhibits oxidative stress and apoptosis could enhance the survival of random skin flaps.

Traditional Chinese Medicine is widely used in China to treat ischemic diseases (Sun L. et al., 2018). Sal B is a water-soluble compound purified from *Salvia miltiorrhiza*, a well-known source material used in TCM (Cao et al., 2012; Li and Wang, 2017). Sal B stimulates bone-marrow angiogenesis by inducing peroxisome proliferator-activated receptor γ (PPARγ) and Runx2 in prednisone-treated rats (Li and Wang, 2017). Moreover, it reduces oxidative stress and neurocyte apoptosis in rats with cerebral small vessel disease via the STAT3/VEGF signaling pathway (Wang and Hu, 2018). It also induces autophagy and has anti-cancer effects via the AKT/mTOR signaling pathway in hepatocellular carcinoma cells (Gong et al., 2016); ameliorates cerebral IRI by inhibiting TLR4/MyD88 signaling pathway (Wang et al., 2016); and promotes angiogenesis and autophagy in acute myocardial infarction (Lin et al., 2016). However, the effects of Sal B on random-pattern cutaneous flaps are unclear, and the underlying mechanisms of any effects are unknown.

We investigated the effects of Sal B on the survival of random-pattern cutaneous flaps and the underlying mechanism, with a focus on the role of autophagy.

## Materials and Methods

### Animals

Healthy Sprague-Dawley rats (male: 250–300 g) were provided by Wenzhou Medical University (License No. SCXK [ZJ] 2005–0019). The Guide for the Care and Use of Laboratory Animals of the China National Institutes of Health was followed for procedures involving animals. All procedures were approved by the Animal Care and Use Committee of Wenzhou Medical University (wydw 2017-0022). All efforts were made to minimize the suffering of the rats. All rats were euthanized by pentobarbital sodium overdose. Seventy-two rats were randomly separated into control, Sal B, and Sal B + 3-methyladenine groups (*n* = 24 each).

### Reagents and Antibodies

Sal B (C_36_H_30_O_16_; purity, 98.5%) was acquired from Tauto Biotech (Shanghai, China). The H&E Staining Kit, DAB developer, and pentobarbital sodium were purchased from Solarbio Science & Technology (Beijing, China). SOD, GSH, and MDA assay kits were acquired from Jiancheng Technology (Nanjing, China). The primary antibody against cadherin 5 was acquired from Boster Biological Technology (A02632-2; Wuhan, China). The primary antibody against GAPDH was purchased from Biogot Technology (AP0063; Shanghai, China). Primary antibodies against VEGF, SOD1, VPS34, MMP9, HO1, CTSD, and CAPS3 were acquired from Proteintech Group (19003-1, 10269-1, 12452-1, 10375-2, 10701-1, 21327-1, and 19677-1; Chicago, IL, United States). Primary antibodies against CYC, Bax, and eNOS were purchased from Cell Signaling Technology (12994, 14796, and 32027; Beverly, MA, United States). The primary antibody against SQSTM1/p62 was purchased from Abcam (ab56416; Cambridge, United Kingdom). Primary antibodies against LC3B and 3MA were purchased from Sigma-Aldrich Chemical Company (L7543 and M9281; Milwaukee, WI, United States). HRP-conjugated IgG secondary antibody was purchased from Santa Cruz Biotechnology (Dallas, TX, United States). FITC-conjugated IgG secondary antibody was obtained from Boyun Biotechnology (Nanjing, China), and DAPI solution was purchased from Beyotime Biotechnology (Jiangsu, China). The BCA Kit was acquired from Thermo Fisher Scientific (Rockford, IL, United States). The ECL Plus Reagent Kit was purchased from PerkinElmer Life Sciences (Waltham, MA, United States).

### Animal Model

Rats were anesthetized by intraperitoneal injection of 40 mg/kg pentobarbital sodium 2% (w/v). The modified McFarlane flap model in the rat dorsum (in the same position in each rat) was performed as previously reported (Kailiang et al., 2016). A caudal skin/panniculus carnosus flap of 3 cm × 9 cm was outlined on the back of the rat, and the skin flap was separated from the underlying fascia. Next, the right and left sacral arteries were excised. Finally, the flap was sutured to the donor bed using 4-0 silk and a wedged-on cutting needle. The random skin flap area was separated into the proximal (area I), intermediate (area II), and distal (area III) zones, each of equal size.

### Group Assignment and Drug Administration

Rats in the Sal B group received an intraperitoneal 40 mg/kg/day injection of Sal B daily. Those in the Sal B + 3MA group received an intraperitoneal injection of 40 mg/kg/day Sal B and 0.5 mg/kg/day 3MA daily. The control group (*n* = 24) received an equal volume of saline daily. The injections were performed from 7 days preoperatively until the rats were euthanized. The rats were housed individually in standard experimental cages in an environment-controlled room and were provided with standard rat chow and water *ad libitum*. All animals were euthanized by pentobarbital sodium overdose at 7 days.

### General Observation and Flap Survival Assessment

Macroscopic changes in flaps were noted for 7 days after the operation; these included appearance, color, texture, and hair condition. Survival area was evidenced by pink and soft skin with new hair growth, whereas necrotic area was evidenced by scabbing, hardening, and dark nidus without new hair growth. At post-operative days 3 and 7, flap survival areas were measured using superimposition of photographs on graph paper, and the percentages were calculated by ImageJ software (National Institutes of Health, Bethesda, MD, United States) as: extent of viable area × 100% ÷ total area.

### Laser Doppler Blood Flow Measurement

Laser Doppler blood flow measurements were performed to evaluate the blood supply to the flaps. At day 7 post-operatively, six rats per group were anesthetized and scanned using a Laserflo BPM (Vasamedic, Saint Paul, MN, United States), with 15 cm × 15 cm area and 256 × 256 pixels. The LDBF protocol was as previously described (Xu et al., 2017). LDBF generally offers deeper penetration enabling enhanced visualization of small vessels below the tissue surface, perfect for angiogenesis evaluation. Blood supply was visualized using the LDBF strong signal (green, yellow, and red), the area of which was quantified using ImageJ software (NIH, Bethesda, MD, United States). The percentage of the flap area with a blood supply was calculated as: strong signal area × 100% ÷ total area.

### Tissue Edema

Tissue edema was assessed by measuring the water content of the flaps. On day 7 post-operatively, flap tissues were harvested, and dehydrated in an autoclave at 50°C. The specimens were weighed daily until constant weight over 2 days. The water content was determined as ([wet weight – dry weight] ÷ wet weight) × 100%.

### Hematoxylin and Eosin Staining

After the rats had been euthanized, three samples (0.5 cm × 0.5 cm) of central tissue from area II were collected, post-fixed in 4% (v/v) paraformaldehyde for 1 day, and embedded in paraffin wax for transverse sectioning. The sections (4 μm thickness) were mounted on poly L-lysine-coated slides for H&E staining. Under a light microscope (20× magnification), the thickness of granulation tissue, edema, and angiogenesis were observed. In addition, the number of microvessels per unit area (/mm^2^) was counted manually.

### SOD Activity, GSH Level, and MDA Content

SOD, GSH, and MDA assays were conducted to evaluate the oxidative stress levels of ischemic flaps. At day 7 post-operatively, tissue samples (0.5 cm × 0.5 cm) were separated from the middle area of area II flaps (*n* = 6 per group), weighed, homogenized, and diluted to 10% (v/v) in an ice bath. The homogenate was centrifuged at 3,500 rpm for 15 min and the supernatant was harvested and its SOD activity (xanthine oxidase method), GSH level (modified 5,5′-dithiobis [2-nitrobenzoic acid] method), and MDA content (modified thiobarbituric acid test) were determined as previously described.

### Immunohistochemistry (IHC)

Six sections of the middle part of area II of the flaps were deparaffinized in xylene and rehydrated through a graded ethanol series. After washing, the sections were blocked in 3% (v/v) H_2_O_2_ and treated with 10.2 mM sodium citrate buffer for 20 min at 95°C. After blocking in 10% (w/v) bovine serum albumin for 10 min, the sections were incubated with antibodies against CD34 (1:100) VEGF (1:300), cadherin 5 (1:100), caspase 3 (1:200), and SOD1 (1:100) overnight at 4°C. Next, the sections were incubated with an HRP-conjugated secondary antibody and counterstained with hematoxylin. Flap tissues were imaged at 20× magnification using the DP2-TWAN image-acquisition system (Olympus, Corp., Tokyo, Japan). Images were analyzed using Image-Pro Plus software (Media Cybernetics, Rockville, MD, United States) for the integral absorbance quantification of VEGF-, cadherin 5-, caspase 3-, SOD1-, and CTSD-, and CD34-positive blood vessels. Six random fields of three random sections of each tissue sample were quantified.

### Immunofluorescence

Six sections of area II were deparaffinized in xylene and rehydrated through a graded ethanol series. After washing, the sections were treated with 10.2 mM sodium citrate buffer for 20 min at 95°C, and permeabilized with 0.1% (v/v) PBS-Triton X-100 for 30 min. After blocking in 10% (v/v) bovine serum albumin in PBS for 1 h, the slides were incubated at 4°C overnight with a primary antibody against LC3 (1:200). Next, the slides were washed three times for 10 min each at room temperature and incubated with FITC-conjugated secondary antibody for 1 h at room temperature. The slides were visualized under a fluorescence microscope (Olympus). The percentage of LC3-positive cells in the dermal layer was determined by counting in six random fields of three random sections from each tissue sample.

### Western Blotting

After the rats had been euthanized, samples (0.5 cm × 0.5 cm) from the middle of area II flaps (*n* = 6) were separated for Western blot analyses. After homogenization, the protein concentration was determined using the BCA assay. Proteins (60 μg) were separated in 12% (w/v) gels and transferred to polyvinylidene difluoride membranes (Roche Applied Science, Indianapolis, IN, United States). After blocking in 10% (w/v) non-fat milk for 2 h, the membranes were incubated with the following primary antibodies at 4°C overnight: VEGF (1:1,000), MMP9 (1:1,000), cadherin 5 (1:1,000), HO1 (1:1,000), eNOS (1:1,000), SOD1 (1:1,000), Bax (1:1000), CYC (1:1,000), caspase 3 (1:1,000), Beclin1 (1:1,000), p62 (1:1,000), LC3 (1:500), VPS34 (1:1,000), CTSD (1:1,000), and GAPDH (1:1,000). The membranes were incubated with the HRP-conjugated IgG secondary antibody (1:5,000) for 2 h at room temperature. Bands were visualized using the ECL Plus Reagent Kit and band intensity was quantified by Image Lab 3.0 software (Bio-Rad, Hercules, CA, United States).

### Statistical Analyses

Statistical analyses were conducted using SPSS ver. 19 software (Chicago, IL, United States). Data are presented as means ± SEM. Comparisons of means between two groups were performed using the Student’s *t*-test. *p*-Values less than 0.05 were considered statistically significant.

## Results

### Effects of Sal B on the Skin Flaps

**FIGURE 1:**
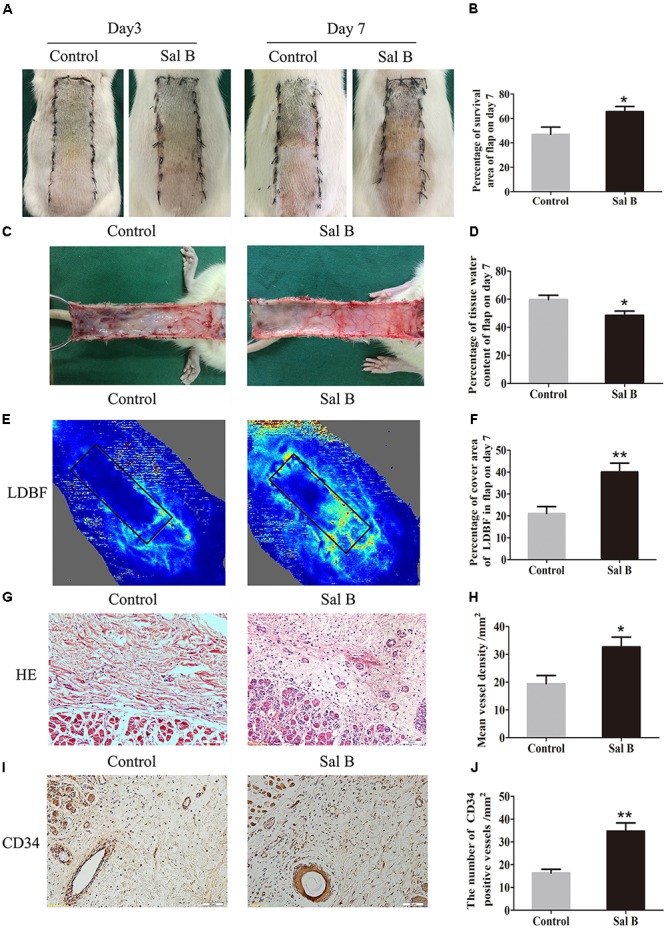
Effects of Sal B on random skin flaps vitality. **(A)** Digital photographs of flap survival of the Control and Sal B group on post-operative days 3 and 7 (scale bar: 2 cm). **(B)** Histogram of percentages of flap survival area on post-operative day 7. **(C)** Digital photographs of the inner side of skin flaps in each group on post-operative day 7 showing tissue edema (scale bar: 2 cm). **(D)** Histogram of percentages of tissue water content of skin flaps. **(E)** LDBF imaging showing vascular flow and blood supply in skin flaps on post-operative day 7 (scale bar: 2 cm). **(F)** Histogram of percentages of area of blood supply. **(G)** H&E staining of microvessels in area II of skin flaps (original magnification, 200×; scale bar: 50 μm). **(H)** Histogram of MVDs by H&E staining. **(I)** IHC for CD34 in area II of skin flaps (original magnification: 200×; scale bar: 50 μm). **(J)** Histogram of CD34-positive vessel densities. ^∗^*p* < 0.05 and ^∗∗^*p* < 0.01 vs. the control group. Data are means ± SEM (*n* = 6 per group).

On day 3, area III of the flaps exhibited necrosis, as evidenced by a dark and brown nidus, which tended to spread to area II. There were no significant differences in the flap survival area between the two groups (Figure 1A). On day 7 in both groups, area I survived, whereas the necrosis in area III had darkened and spread to area II, with scabbing and hardening (Figure 1A). The Sal B group showed a significantly larger mean survival area than the control group (65.66 ± 10.20 and 46.94 ± 14.62%, respectively; *p* = 0.028; Figure 1B). In the control group, the distal part of the flaps was swollen and bruised, with subcutaneous venous blood stasis (Figure 1C). These signs were less apparent in the Sal B group. The water content of tissue was significantly lower in the Sal B group (48.49 ± 7.34%) than in the control group (59.72 ± 7.62%; *p* = 0.027; Figure 1D). Moreover, the Sal B group showed a greater area of blood flow than the control group (Figure 1E). The area of vascular flow was significantly larger in the Sal B group than in the control group (40.08 ± 9.86 and 21.08 ± 7.71%, respectively; *p* = 0.004; Figure 1F). Flaps in the Sal B group contained a greater number of microvessels than the control group (Figure 1G). The MVD of area II in the Sal B group was 32.69 ± 8.61/mm^2^, significantly greater than that in the control group (19.41 ± 7.31/mm^2^; *p* = 0.016; Figure 1H). Sal B treatment increased the number of CD34-positive vessels (34.73 ± 8.91/mm^2^ in the Sal B group and 16.34 ± 4.06/mm^2^ in the control group; *p* = 0.001; Figures 1I,J).

### Sal B Promotes Angiogenesis in Areas of Ischemia

**FIGURE 2:**
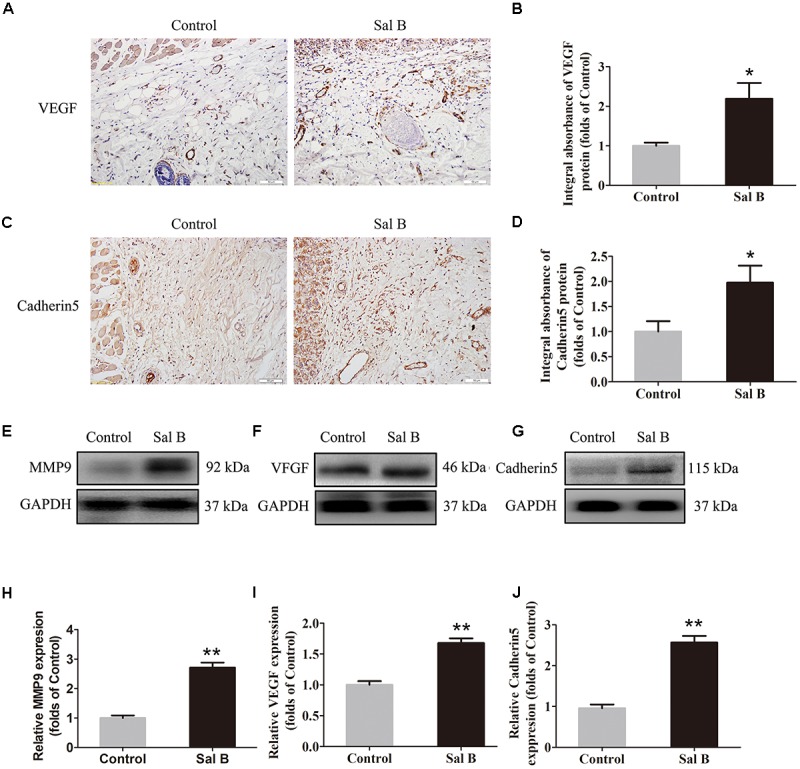
Sal B promotes angiogenesis in ischemic random skin flaps. Rats were killed and the samples in middle part of flap area II were harvested for analysis on post-operative day 7. **(A,C)** IHC of VEGF and cadherin 5 expression of skin flaps in the Control and Sal B group (original magnification: 200×; scale bar: 50 μm). **(B**,**D)** Histograms of optical density values of VEGF and cadherin 5 by IHC, respectively. **(E**–**G)** Western blotting of MMP9, VEGF, and cadherin 5 levels of skin flaps in the Control and Sal B group, corrected by GAPDH as internal control. The gels have been run under the same experimental conditions. The original images are available in Supplementary Figure S1A. **(H**–**J)** Histograms of optical density values of MMP9, VEGF, and cadherin 5 in the two groups as determined by Western blotting. ^∗^*p* < 0.05 and ^∗∗^*p* < 0.01 vs. the control group. Data are means ± SEM (*n* = 6 per group).

Vascular endothelial growth factor, MMP9, and cadherin 5 levels were evaluated by IHC. As shown in Figure 2A, VEGF was detected in vessels and stromal cells in area II, and at a higher level in the Sal B group than in the control group (*p* = 0.015; Figure 2B). Cadherin 5 expression in vessels and stromal cells (Figure 2C) was also higher in the Sal B group than in the control group (*p* = 0.034; Figure 2D). Western blotting showed that MMP9, VEGF, and cadherin 5 levels were significantly higher in the Sal B group than in the control group (*p* = 0.001, 0.001, and <0.001, respectively; Figures 2E–J).

### Sal B Reduces Apoptosis in Areas of Ischemia

**FIGURE 3:**
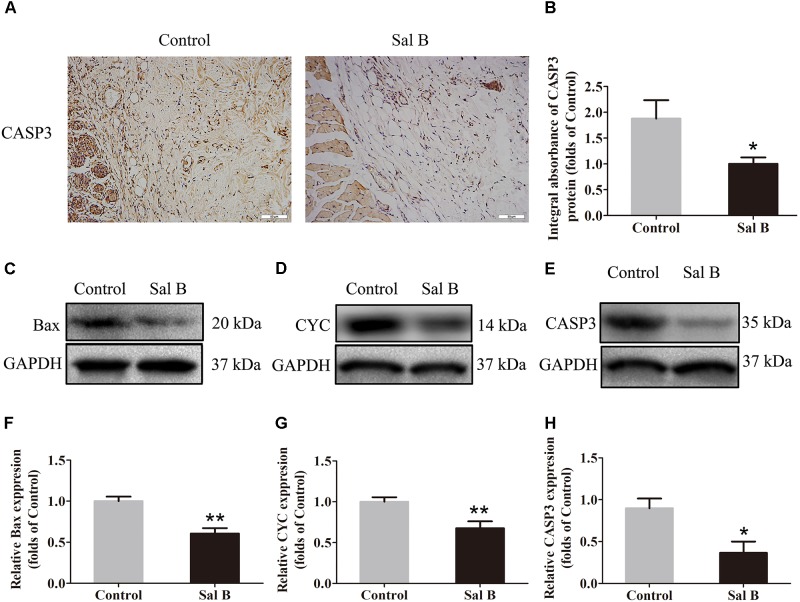
Sal B inhibits apoptosis in random skin flaps. Rats were killed and the samples in middle part of flap area II were harvested for analysis on post-operative day 7. **(A)** Caspase 3 expression in skin flaps of the Control and Sal B groups, evaluated by IHC (original magnification: 200×; scale bar: 50 μm). **(B)** Histogram of optical density values of Caspase 3 expression by IHC. **(C**–**E)** Western blotting for protein levels of Bax, CYC, and caspase 3, respectively, corrected by GAPDH as internal control. The gels have been run under the same experimental conditions. The original images are available in Supplementary Figure S1B. **(F**–**H)** Histograms of optical density values of Bax, CYC, and caspase 3 as determined by Western blotting. ^∗^*p* < 0.05 and ^∗∗^*p* < 0.01 vs. the control group. Data are means ± SEM (*n* = 6 per group).

Caspase 3 levels in the dermis layer of flap area II were lower in the Sal B group than in the control group, as determined by IHC (Figure 3A). The integral absorbance of caspase 3 in the Sal B group was significantly lower than that in the control group (*p* < 0.044) (Figure 3B). Bax, CYC, and caspase 3 levels were also determined by Western blotting (Figures 3C–E, respectively). The CYC level was significantly higher in the control group than in the Sal B group (*p* = 0.001; Figure 3F). However, Bax and caspase 3 levels were lower in the Sal B group than in the control group (*p* = 0.001 and 0.013, respectively; Figures 3G,H).

### Sal B Reduces Oxidative Stress in Areas of Ischemia

**FIGURE 4:**
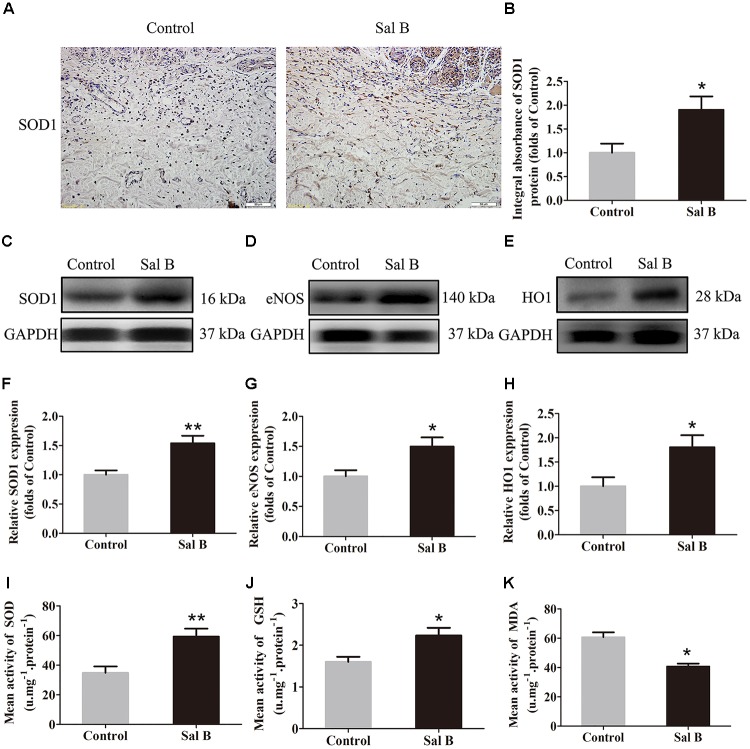
Sal B reduces oxidative stress in random skin flaps. Rats were killed and the samples in middle part of flap area II were harvested for analysis on post-operative day 7. **(A)** SOD1 expression in skin flaps of the Control and Sal B groups, assessed by IHC (original magnification: 200×; scale bar: 50 μm). **(B)** Histogram of optical density values of SOD1 by IHC. **(C–E)** Western blotting for protein levels of SOD1, eNOS, and HO1, respectively, corrected by GAPDH as internal control. The gels have been run under the same experimental conditions. The original images are available in Supplementary Figure S1C. **(F–H)** Histograms of optical density values of SOD1, eNOS, and HO1 as determined by Western blotting. **(I)** Total SOD activity by xanthine oxidase method. **(J)** GSH level by modified 5,5′-dithiobis [2-nitrobenzoic acid] method. **(K)** MDA level by modified thiobarbituric acid test. ^∗^*p* < 0.05 and ^∗∗^*p* < 0.01 vs. the control group. Data are means ± SEM (*n* = 6 per group).

SOD, SOD1, eNOS, HO1, GSH, and MDA levels were determined to evaluate the magnitude of oxidative stress in the flaps. SOD1 expression (by IHC) and integral absorbance in the dermis were higher in the Sal B group than in the control group (*p* = 0.024; Figures 4A,B). Furthermore, Western blotting indicated that SOD1, eNOS, and HO1 levels (Figures 4C–E, respectively) were significantly higher in the Sal B group than in the other groups (*p* = 0.004, 0.022, and 0.026, respectively; Figures 4F–H). The Sal B group had a higher mean SOD level (59.33 ± 13.20 U mg^-1^ protein^-1^) than the control group (34.83 ± 10.65 U mg^-1^ protein^-1^; *p* = 0.005; Figure 4I). The mean GSH level in the Sal B group was 2.23 ± 0.45 nM mg^-1^ protein^-1^ compared to 1.60 ± 0.30 nM mg^-1^ protein^-1^ in the control group (*p* = 0.018; Figure 4J). The mean MDA level in the Sal B group was 40.67 ± 5.05 nM mg^-1^ protein^-1^ compared to 60.67 ± 8.31 nM mg^-1^ protein^-1^ in the control group (*p* = 0.001; Figure 4K).

### Sal B Activates Autophagy in Areas of Ischemia

**FIGURE 5:**
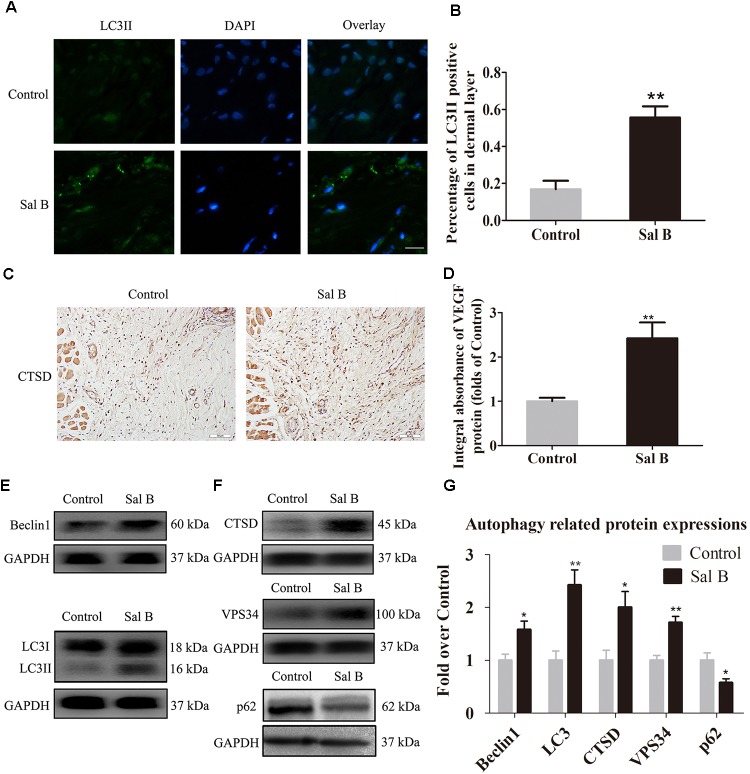
Sal B activates autophagy in random skin flaps. Rats were killed and the samples in middle part of flap area II were harvested for analysis on post-operative day 7. **(A)** Autophagy level in skin flaps were estimated by immunofluorescence staining of LC3II: autophagosomes (green) in the dermis in area II in each group; nuclei counterstained with DAPI (blue) (scale bar: 15 μm). **(B)** Histogram of frequency of LC3II-positive cells. **(C)** CTSD expression by IHC (original magnification: 200×; scale bar: 50 μm). **(D)** CTSD levels in random skin flaps as estimated by Western blotting. **(E,F)** Beclin1, LC3II, CTSD, VPS34, and p62 levels by Western blotting, respectively, corrected by GAPDH as internal control. The gels have been run under the same experimental conditions. The original images are available in Supplementary Figure S1D. **(G)** Histograms of optical density values of Beclin1, LC3II, CTSD, VPS34, and p62 expressions as determined by Western blotting. ^∗^*p* < 0.05 and ^∗∗^*p* < 0.01 vs. the control group. Data are means ± SEM (*n* = 6 per group).

Beclin1 and VPS34 are localized to the pre-autophagosomal compartment, and LC3II is present in the autophagosome membrane. Therefore, we analyzed Beclin1, VPS34, and LC3II levels as markers of autophagosomes, that of CTSD as a marker of autolysosomes, and that of p62 to monitor autophagic degradation (Figure 5A). The frequency of LC3II-positive cells in the dermis in the Sal B group was higher than that in the control group (Figure 5B). The CTSD level and integral absorbance in the dermis were higher in the Sal B group than in the control group (*p* = 0.003; Figures 5C,D). Furthermore, Western blotting showed that Beclin1, LC3II, VPS34, CTSD, and p62 levels (Figures 5E–G) were significantly higher in the Sal B group than in the control group (*p* = 0.015, 0.002, 0.001, and 0.018, respectively), whereas that of p62 was lower in the Sal B group (*p* = 0.024).

### Inhibition of Autophagy Reverses the Effects of Sal B

**FIGURE 6:**
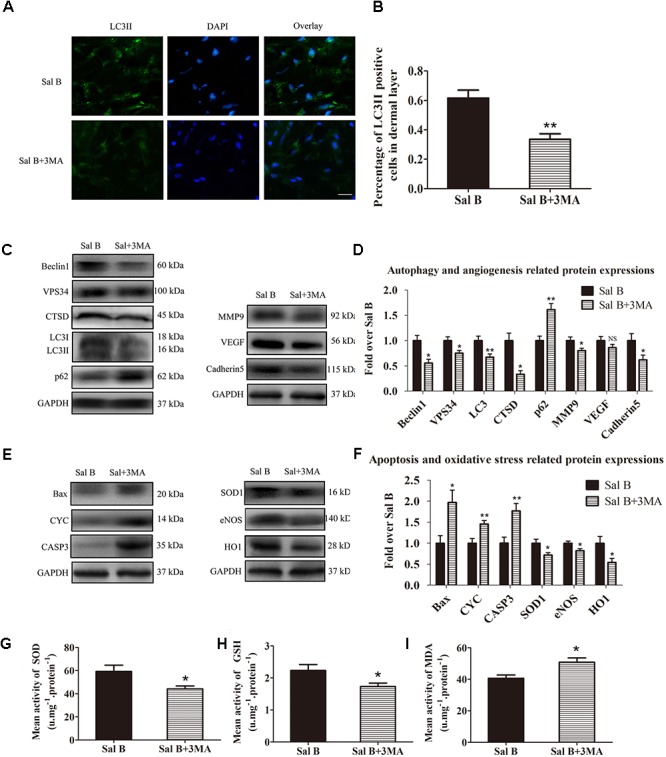
Inhibition of autophagy reverses the effects of Sal B in random skin flaps. Rats were killed and the samples in middle part of flap area II were harvested for analysis on post-operative day 7. **(A)** Autophagy level in skin flaps were estimated by immunofluorescence staining of LC3II:Autophagosomes (green) in the dermis in area II in each group; nuclei counterstained with DAPI (blue) (scale bar: 15 μm). **(B)** Histogram of frequency of LC3II-positive cells in the dermis. **(C)** Western blotting of Beclin1, VPS34, CTSD, LC3II, and p62 levels, as well as those of MMP9, VEGF, and cadherin 5, corrected by GAPDH as internal control. The gels have been run under the same experimental conditions. The original images are available in Supplementary Figures S2A,B. **(D)** Histograms of optical density values of autophagy- (Beclin1, VPS34, CTSD, LC3II, and p62) and angiogenesis- (MMP9, VEGF, and cadherin 5) related proteins, assessed by Western blotting. **(E)** Western blotting of Bax, CYC, and caspase 3 levels, as well as those of SOD1, eNOS, and HO1, corrected by GAPDH as internal control. The original images are available in Supplementary Figures S2C,D. **(F)** Histograms of optical density values of apoptosis- (Bax, CYC, and caspase 3) and oxidative stress-related proteins (Bax, CYC, and caspase 3) as determined by Western blotting. **(G)** Total SOD activity by xanthine oxidase method. **(H)** GSH level by modified 5,5′-dithiobis [2-nitrobenzoic acid] method. **(I)** MDA level by modified thiobarbituric acid test. ^∗^*p* < 0.05 and ^∗∗^*p* < 0.01 vs. the control group. Data are means ± SEM (*n* = 6 per group); NS, not significant.

**FIGURE 7:**
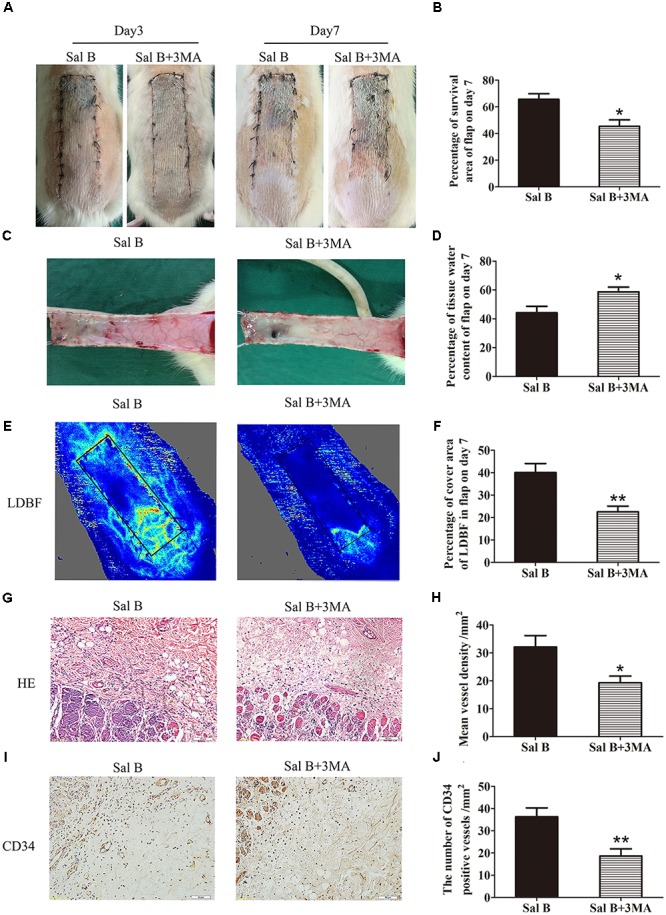
Inhibition of autophagy reverses the effects of Sal B on flap vitality. **(A)** Digital photographs of flap survival of the Control and Sal B group on post-operative days 3 and 7 (scale bar: 2 cm). **(B)** Histogram of percentages of flap survival area on post-operative day 7. **(C)** Digital photographs of the inner side of skin flaps in each group on post-operative day 7 showing the degree of edema (scale bar: 2 cm). **(D)** Histogram of percentages of tissue water content of skin flaps. **(E)** LDBF imaging showing vascular flow and blood supply in skin flaps on post-operative day 7 (scale bar: 2 cm). **(F)** Histogram of percentages of area of blood supply. **(G)** H&E staining of microvessels in area II of skin flaps (original magnification: 200×; scale bar: 50 μm). **(H)** Histogram of MVDs as determined by H&E staining. **(I)** IHC for CD34 in area II of skin flaps (original magnification: 200×; scale bar: 50 μm). **(J)** Histogram of CD34-positive vessel densities. ^∗^*p* < 0.05 and ^∗∗^*p* < 0.01 vs. the control group. Data are means ± SEM (*n* = 6 per group).

Immunofluorescence staining of autophagosomes in area II was performed (Figure 6A). The frequency of LC3II-positive cells in the dermis in the Sal B group was greater than that in the Sal B + 3MA group (*p* = 0.002; Figure 6B). Western blotting indicated that Beclin1, VPS34, CTSD, and LC3II levels were significantly higher in the Sal B group than in the Sal B + 3MA group (*p* = 0.034, 0.022, 0.002, and 0.013, respectively; Figures 6C,D), as were those of MMP9 and cadherin 5 (*p* = 0.043 and 0.047, respectively; Figures 6C,D) and SOD1, eNOS, and HO1 (*p* = 0.030, 0.028, and 0.034, respectively; Figures 6E,F) but not VEGF (*p* = 0.209; Figures 6C,D). By contrast, the p62, CYC, Bax, and caspase 3 levels were significantly lower in the Sal B group than in the Sal B + 3MA group (*p* = 0.002, 0.008, 0.018, and 0.008, respectively; Figures 6E,F). The mean SOD level was higher in the Sal B group (59.33 ± 13.20 U mg^-1^ protein^-1^) than in the Sal B + 3MA group (44.17 ± 6.37 U mg^-1^ protein^-1^; *p* = 0.030; Figure 6G). The mean GSH level in the Sal B group was 2.23 ± 0.45 nM mg^-1^ protein^-1^ compared to 1.73 ± 0.27 nM mg^-1^ protein^-1^ in the Sal B + 3MA group (*p* = 0.042; Figure 6H). The mean MDA level in the Sal B group was 40.67 ± 5.05 nM mg^-1^ protein^-1^ compared to 50.83 ± 6.70 nM mg^-1^ protein^-1^ in the Sal B + 3MA group (*p* = 0.014; Figure 6I). On day 3, there were no significant differences in the flap survival area between the Sal B and Sal B + 3MA groups (Figure 7A). On day 7, the necrosis of flaps became darker and the Sal B group had a significantly larger mean survival area than the Sal B + 3MA group (65.66 ± 10.20 and 45.42 ± 11.85%, respectively; *p* = 0.010; Figure 7B). Figure 7C shows edema on the inner side of the flaps. The water content of tissue was significantly lower in the Sal B group (48.49 ± 7.34%) than in the Sal B + 3MA group (58.72 ± 7.92%; *p* = 0.043; Figure 7D). The Sal B group had a larger blood flow area than the Sal B + 3MA group (Figure 7E). The percentage LDBF area was significantly greater in the Sal B group than in the Sal B + 3MA group (40.08 ± 9.86 and 22.57 ± 6.20%, respectively; *p* = 0.004; Figure 7F). In addition, H&E staining showed that the Sal B group had a greater number of microvessels than the Sal B + 3MA group (Figure 7G). The MVD of area II in the Sal B group was significantly higher than that in the Sal B + 3MA group (32.69 ± 8.61/mm^2^ and 19.34 ± 5.85/mm^2^, respectively; *p* = 0.023; Figure 7H). Moreover, 3MA decreased the number of CD34-positive vessels (34.73 ± 8.91/mm^2^ in the Sal B + 3MA group, compared with the Sal B group (18.67 ± 7.87/mm^2^; *p* = 0.006; Figures 7I,J).

## Discussion

Sal B, an extract of *S. miltiorrhiza*, is used for the treatment of various vascular diseases involving the cardiovascular system (Lin et al., 2016), cerebral small blood vessels (Wang and Hu, 2018), and osteonecrosis of the femoral head in TCM (Li and Wang, 2017). Ischemia and necrosis in the distal part of random skin flaps due to a reduced blood supply are commonly encountered in plastic and reconstructive surgery. However, evidence-based data on the efficacy of Sal B in random-pattern skin flaps are limited. Our data suggest that Sal B promotes the survival of random skin flaps by inducing autophagy and angiogenesis, as well as attenuating oxidative stress and apoptosis.

Sal B enhances angiogenesis in murine SVR endothelial cells *in vitro* (Lay et al., 2003), as well as in animal models of myocardial infarction (Lin et al., 2016). In our study, H&E and IHC staining for CD34 showed that Sal B significantly increased the number of microvessels in the dermis of ischemic flaps. Moreover, vascular flow was greater in the Sal B group than in the control group. These findings indicate that Sal B promotes the survival of random skin flaps by increasing their blood supply. Angiogenesis involves destruction of preexisting cell connections, mitosis, sprouting of endothelial cells, and maturation of new capillaries (Nowak-Sliwinska et al., 2018). MMP9 degrades proteins related to vessel wall stability (Li et al., 2018), VEGF induces mitosis of endothelial cells (Stacker et al., 2016), and cadherin 5 forms intercellular junctions. Western blotting and IHC indicated that Sal B increased VEGF and cadherin 5 expression in the dermis of ischemic flaps. Furthermore, MMP9 levels were higher in the Sal B group than in the control group. Thus, Sal B promotes angiogenesis in the dermis of random skin flaps.

In random skin flaps, post-ischemic reperfusion induces oxidative stress and apoptosis, leading to tissue damage (Kosuru et al., 2018). In the early stages of oxidative stress, ROS react with cell membrane lipids and proteins, triggering peroxidation and destruction of cells (Anet et al., 2018). As a marker of lipid peroxidation, the MDA level reflects the extent of tissue injury (Tsikas, 2017). Furthermore, SOD and GSH are indicators of antioxidant status (He et al., 2016), eNOS has antioxidant activity (Sun M.S. et al., 2018; Yu and Liu, 2018), and HO1 is expressed during the oxidative stress response (Fuyuno et al., 2018). Sal B promotes functional recovery from cerebral vessel disease by reducing oxidative stress (Wang and Hu, 2018). Western blotting and IHC indicated that Sal B increased SOD1, eNOS, and HO1 expression in the dermis of ischemic flaps. Moreover, it increased the SOD and GSH levels, and decreased the MDA level, in ischemic flaps. In short, Sal B attenuates the level of oxidative stress in random skin flaps.

Sal B reportedly prevents apoptosis in various ischemic diseases such as hepatic IRI, and acute myocardial infarction (Kong et al., 2009; Lin et al., 2016). The pathologic mechanism of necrosis of ischemic flaps after post-ischemic perfusion involves apoptosis (Ren et al., 2018). Therefore, we hypothesized that Sal B inhibits apoptosis in areas of ischemia, which promotes the survival of random-pattern skin flaps. Thus, we analyzed apoptosis in areas of ischemia in the skin flaps. Mitochondria are centrally located during apoptosis (Zhang et al., 2018). Bax induces permeabilization of the mitochondrial outer membrane, leading to swelling (Wei et al., 2018). CYC is released from mitochondria and forms an apoptosome, which activates caspase 3 to induce apoptosis (Zhang et al., 2018). Hence, Bax, CYC, and caspase 3 levels were determined to analyze the magnitude of apoptosis. The caspase 3 level in area II was markedly decreased by Sal B. Moreover, Western blotting showed that Bax and CYC levels were significantly lower in the Sal B group than in the control group. Therefore, Sal B inhibits apoptosis in random skin flaps.

Autophagy is a natural, regulated mechanism for the degradation of unnecessary or dysfunctional components such as cytosolic macromolecules and organelles, and it protects against metabolic stress (Jia et al., 2018; Scrivo et al., 2018). In disease, autophagy is an adaptive response to stress, which promotes survival, whereas in other situations it promotes cell death (Giansanti et al., 2011). Autophagy is important in vascular disease including acute myocardial infarction and cerebral small blood vessel disease (Lin et al., 2016; Wang and Hu, 2018). Furthermore, Sal B reportedly promotes autophagy in vascular disease (Li et al., 2016). However, the role of autophagy in random skin flaps is unclear. To the best of our knowledge, this is the first study on the effects of Sal B on autophagy in random skin flaps. Autophagy involves autophagosome formation, autolysosome formation, and substrate degradation. Therefore, we analyzed Beclin1, VPS34, and LC3II as markers of autophagosomes (Yang et al., 2018), CTSD as a marker of autolysosomes (Wu et al., 2017), and p62 as a marker of autophagic degradation (Zaffagnini and Martens, 2016; Zaffagnini et al., 2018). Immunofluorescence revealed that LC3II and CTSD expression were greater in the Sal B group. Moreover, Beclin1, LC3II, and VPS34 expression were upregulated, which indicates an increase in the number of autophagosomes in areas of ischemia. Next, we assessed autophagic degradation by evaluating p62 and CTSD levels. The CTSD level was higher, and that of p62 was lower, in the Sal B group than in the control group, which indicates enhanced autophagic flux in the Sal B group. Therefore, Sal B activates autophagy in random skin flaps.

We also evaluated the role of Sal B-induced autophagy in random skin flaps. Inhibition of autophagy by 3MA reversed the Sal B-induced increase in survival, reduction of tissue edema, and increase in microvessel density. Autophagy promotes angiogenesis by activating AKT phosphorylation in endothelial cells (Perez-Alvarez et al., 2018). We found that administration of 3MA significantly reduced MMP9 and cadherin 5 levels. Therefore, Sal B promotes angiogenesis in random skin flaps by activating autophagy. Autophagy also degrades polyubiquitinated proteins and damaged mitochondria to prevent ER stress-induced mitochondria-mediated apoptosis (Tang et al., 2011; Lee et al., 2015). In present study, Bax, CYC, and caspase 3 levels were decreased by 3MA, which suggests that the anti-apoptosis effects of Sal B are mediated by activation of autophagy. In addition, autophagy is required to maintain healthy mitochondria to prevent the release of ROS from damaged mitochondria (López et al., 2014). In this study, 3MA significantly decreased the SOD, SOD1, eNOS, HO1, and GSH levels, and increased that of MDA, in Sal B-treated flaps. Thus, Sal B reduces oxidative stress in random skin flaps by activating autophagy.

However, it is no doubt that the limitations of Sal B in the random skins therapy still need to be further investigated. For example, There are many active molecules and pharmaceuticals, such as VEGF(165), calcitriol, curculigoside A, have been shown to increase the survival of random pattern skin flaps in rats (Giunta et al., 2005; Zhou et al., 2016; Chen et al., 2018). However, none of them have come to clinical application. Application in large animal model (proof of concept as a preclinical study) will be beneficial in these studies. Therefore, for further translational research, rabbit or pig will be used to verify the beneficial effect of Sal B on the flaps survival. Moreover, the Sal B treatment used 7 days before the surgery is also hard to apply clinically. Therefore, the time of Sal B administration for the flap survival promotion beginning after the surgery is needed to be investigated.

## Conclusion

Sal B has pro-angiogenesis, anti-apoptosis, and anti-oxidative stress effects by promoting autophagy, which enhances the survival of random-pattern skin flaps.

## Author Contributions

JL wrote the manuscript text. RL, ShL, HW, JD, and GX prepared figures and collected samples. SL, YW, and DL analyzed data. KZ designed the experiment. WG, JK, HX, and KZ revised manuscript. All authors reviewed and approved the final manuscript.

## Conflict of Interest Statement

The authors declare that the research was conducted in the absence of any commercial or financial relationships that could be construed as a potential conflict of interest.

## Supplementary Material

FIGURE S1 Original western blot images for comparison of the Control and Sal B groups. **(A)** The original gel image for Figures 2E–G. **(B)** The original gel images for Figures 3C–E. **(C)** The original gel image for Figures 4C–E. **(D)** The original gel image for Figures 5E,F.

FIGURE S2 Original western blot images for comparison of the Sal B and Sal B + 3MA groups. **(A,B)** The original gel image for Figure 6C. **(B,D)** The original gel images for Figure 6E.

## Abbreviations

BCA: ECL: LDBF: laser doppler blood flow
cadherin5: vascular endothelial cadherin
CAPS3: caspase 3
CTSD: cathepsin D
CYC: cytochrome c
DAPI: 4′,6-diamidino-2-phenylindole
eNOS: endothelial nitric oxide synthase
FITC: fluorescein isothiocyanate
GAPDH: glyceraldehyde 3-phosphate dehydrogenase
GSH: glutathione
H&E: hematoxylin and eosin
HO1: heme oxygenase-1
HRP: horseradish peroxidase
IHC: immunohistochemistry
LC3B: autophagy marker light chain 3B
3MA: 3-methyladenine
MDA: malondialdehyde
MMP9: matrix metallopeptidase 9
mTOR1: mammalian target of rapamycin complex-1
MVD: the mean vessel density
MyD88: myeloid differentiation factor 88
IRI: ischemia-reperfusion injury
PPARγ: peroxisome proliferator-activated receptor γ
Runx2: runt-related transcription factor 2
Sal B: salvianolic acid B
SOD: superoxide dismutase
SQSTM1/p62: nucleoporin p62
STAT3: the signal transducer and activator of transcription 3
TCM: traditional Chinese medicine
TLR4: the Toll-like receptor 4
VEGF: vascular endothelial growth factor
VPS34: phosphoinositide-3-kinase
