# Supplementary figures and images for: Salvianolic Acid B Promotes the Survival of Random-Pattern Skin Flaps in Rats by Inducing Autophagy
Source: Front Pharmacol. 2018 Oct 23;9:1178. doi: 10.3389/fphar.2018.01178 (PMC6206168; doi:10.3389/fphar.2018.01178)

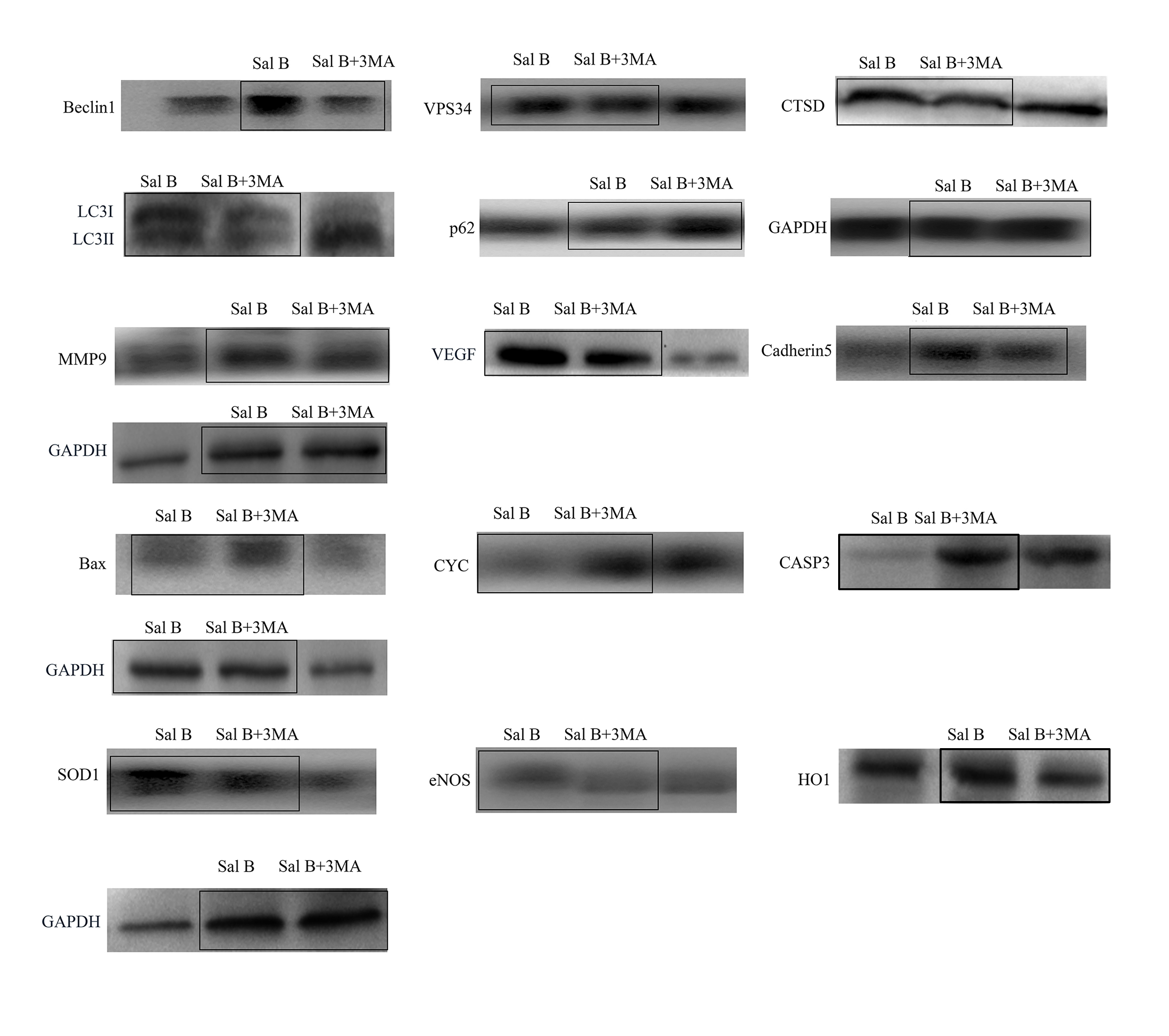

Supplement: FIGURE S1 — Original western blot images for comparison of the Control and Sal B groups. (A) The original gel image for Figures 2E–G. (B) The original gel images for Figures 3C–E. (C) The original gel image for Figures 4C–E. (D) The original gel image for Figures 5E,F. [file Image_1.JPEG]

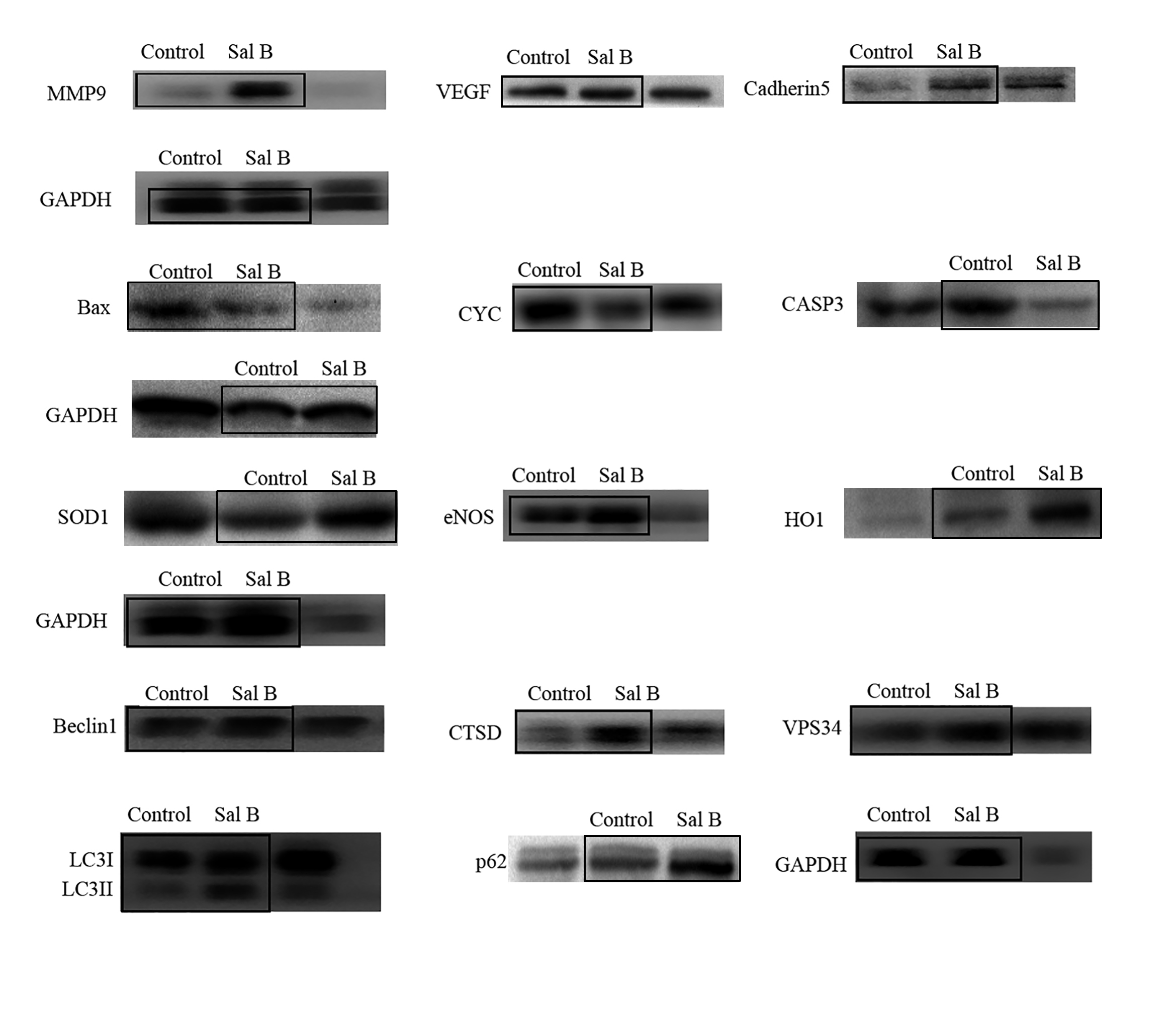

Supplement: FIGURE S2 — Original western blot images for comparison of the Sal B and Sal B + 3MA groups. (A,B) The original gel image for Figure 6C. (B,D) The original gel images for Figure 6E. [file Image_2.JPEG]
